# The Zika Virus Epidemic in Brazil: From Discovery to Future Implications

**DOI:** 10.3390/ijerph15010096

**Published:** 2018-01-09

**Authors:** Rachel Lowe, Christovam Barcellos, Patrícia Brasil, Oswaldo G. Cruz, Nildimar Alves Honório, Hannah Kuper, Marilia Sá Carvalho

**Affiliations:** 1 Department of Infectious Disease Epidemiology, London School of Hygiene & Tropical Medicine, Keppel Street, London WC1E 7HT, UK; 2 Centre for the Mathematical Modelling of Infectious Diseases, London School of Hygiene & Tropical Medicine, Keppel Street, London WC1E 7HT, UK; 3 Barcelona Institute for Global Health (ISGLOBAL), Doctor Aiguader, 88, 08003 Barcelona, Spain; 4 Institute of Health Communication and Information, Oswaldo Cruz Foundation (Fiocruz), Avenida Brasil 4365, Rio de Janeiro 21045-900, Brazil; xris@fiocruz.br; 5 Instituto Nacional de Infectologia Evandro Chagas, Oswaldo Cruz Foundation (Fiocruz), Avenida Brasil 4365, Rio de Janeiro 21045-900, Brazil; patricia.brasil@ini.fiocruz.br; 6 Scientific Computation Program, Oswaldo Cruz Foundation (Fiocruz), Avenida Brasil 4365, Rio de Janeiro 21045-900, Brazil; oswaldo.cruz@fiocruz.br (O.G.C.); marilia.carvalho@fiocruz.br (M.S.C.); 7 Laboratório de Mosquitos Transmissores de Hematozoários, Instituto Oswaldo Cruz (Fiocruz), Avenida Brasil 4365, Rio de Janeiro 21045-900, Brazil; honorio@ioc.fiocruz.br; 8 Núcleo Operacional Sentinela de Mosquitos Vetores-Nosmove/Fiocruz, Avenida Brasil 4365, Rio de Janeiro 21045-900, Brazil; 9 International Centre for Evidence in Disability, London School of Hygiene & Tropical Medicine, Keppel Street, London WC1E 7HT, UK; hannah.kuper@lshtm.ac.uk

**Keywords:** Zika virus, microcephaly, Congenital Zika Syndrome, epidemiology, surveillance, vector control, socio-economic impact

## Abstract

The first confirmed case of Zika virus infection in the Americas was reported in Northeast Brazil in May 2015, although phylogenetic studies indicate virus introduction as early as 2013. Zika rapidly spread across Brazil and to more than 50 other countries and territories on the American continent. The *Aedes* *aegypti* mosquito is thought to be the principal vector responsible for the widespread transmission of the virus. However, sexual transmission has also been reported. The explosively emerging epidemic has had diverse impacts on population health, coinciding with cases of Guillain–Barré Syndrome and an unexpected epidemic of newborns with microcephaly and other neurological impairments. This led to Brazil declaring a national public health emergency in November 2015, followed by a similar decision by the World Health Organization three months later. While dengue virus serotypes took several decades to spread across Brazil, the Zika virus epidemic diffused within months, extending beyond the area of permanent dengue transmission, which is bound by a climatic barrier in the south and low population density areas in the north. This rapid spread was probably due to a combination of factors, including a massive susceptible population, climatic conditions conducive for the mosquito vector, alternative non-vector transmission, and a highly mobile population. The epidemic has since subsided, but many unanswered questions remain. In this article, we provide an overview of the discovery of Zika virus in Brazil, including its emergence and spread, epidemiological surveillance, vector and non-vector transmission routes, clinical complications, and socio-economic impacts. We discuss gaps in the knowledge and the challenges ahead to anticipate, prevent, and control emerging and re-emerging epidemics of arboviruses in Brazil and worldwide.

## 1. Zika Virus Emergence and Spread in Brazil

**Figure 1:**
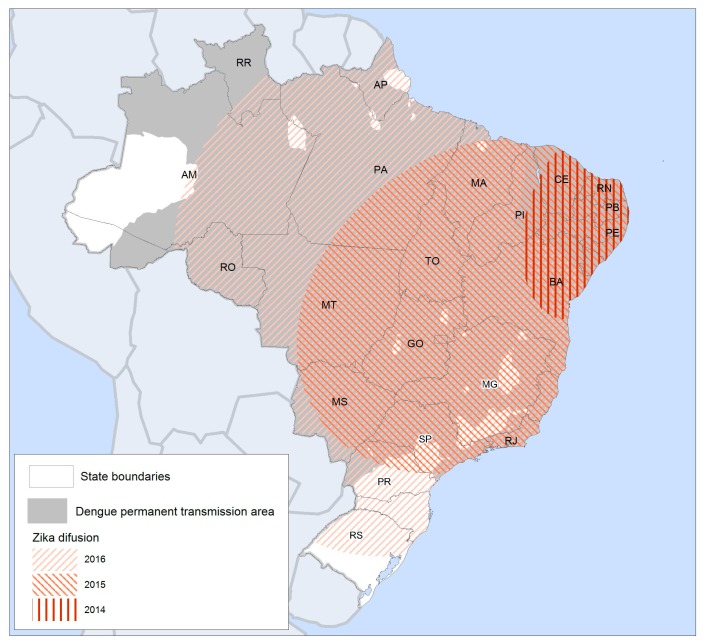
Spatial diffusion of Zika virus (ZIKV) in Brazil from 2014 to 2016 according to case reports and epidemiologic data produced by the Federal Ministry of Health and state secretaries of health [7]. The current permanent dengue transmission area is shown in grey [8].

Zika virus (ZIKV) infection is an acute exanthematous disease transmitted by the same vectors as some of the most significant arthropod-borne viral diseases in the world, including dengue, chikungunya, and yellow fever [1]. The virus circulated silently in Africa and Asia for over half a century with few records describing the clinical presentations of ZIKV infection, which are similar to other arboviral infections. Symptoms include mild fever, rash, arthritis, headache, conjunctivitis, and edema [2]. There is one ZIKV serotype with two ZIKV lineages (African and Asian) and three ZIKV genotypes (West African, East African, and Asian). In 2007, the first ZIKV outbreak was reported in Yap island, Micronesia, followed by epidemics in several Pacific Islands, including French Polynesia, between 2013 and 2014 [3]. An Asian ZIKV genotype is thought to have arrived in Brazil as early as 2013, sharing a common ancestor with the ZIKV strain that circulated during the French Polynesian epidemic [4]. The first reports of an unknown exanthematic disease outbreak in Brazil, later identified as ZIKV infection, were issued in December 2014. In May 2015, the spread of ZIKV among the local population was laboratory confirmed, first in the states of Pernambuco (PE), Rio Grande do Norte (RN), and Bahia (BA) in the Northeast region, then in other states of the Central-West and Southeast regions [5,6]. Figure 1 summarises the spread of the ZIKV epidemic in Brazil according to state and national epidemiological reports.

By 2016, ZIKV had spread to most states, except some remote areas in the Amazon region and the southernmost portion of the country, where the climate is not favourable for the vector. While the ZIKV epidemic spread to a vast area in just a few months, the four dengue virus (DENV) serotypes (DENV1-4), also transmitted by *Aedes* mosquito vectors, took several decades, spreading through coastal and tropical zones between 1980 and 2010 and more recently towards inland areas and higher altitudes. ZIKV transmission also spread beyond the boundaries of permanent DENV transmission (Figure 1), which is limited by a climatic barrier in the south and low population density areas in the north [8,9]. The unknown rate of asymptomatic cases makes it difficult to ascertain true population-level exposure, but a recent serosurvey conducted in Salvador in Northeast Brazil suggested a peak seroprevalence of 63% by 2016 [10]. Overall, the number of reported ZIKV cases in Brazil has decreased from 205,578 cases in 2016 to 13,353 in 2017 (up to epidemiological week 25) [11], with population immunity thought to be the main cause of the decline [12]. However, continued transmission of the four DENV serotypes in Brazil and the Americas over many decades suggests that ZIKV will continue to circulate within the human transmission cycle for the foreseeable future [13].

In this article, we provide an overview of the current knowledge of the ZIKV epidemic from the Brazilian perspective, including the discovery of severe complications related to the virus, vector and non-vector ZIKV transmission routes, clinical features, and the social and economic impact of the disease. We discuss gaps in the knowledge and the challenges ahead in anticipating, preventing, and controlling emerging and re-emerging epidemics of ZIKV and other arboviruses in the future.

## 2. A Public Health Emergency

### 2.1. Building the Evidence

**Figure 2:**
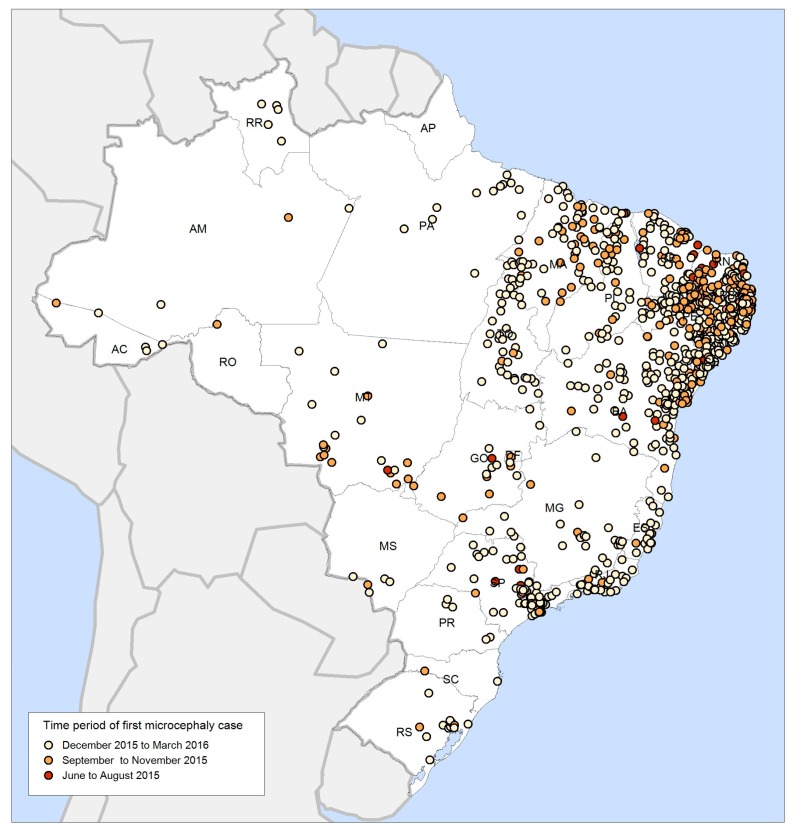
Spatial diffusion of microcephaly in Brazil, June 2015 to March 2016. Data obtained from the public health events registry (RESP) information system, provided to the authors by the Brazilian Ministry of Health.

ZIKV was considered a benign disease until October 2015, when a sharp increase in the number of neonates born with microcephaly, a rare condition associated with incomplete brain development, was observed in maternity services in Northeast Brazil [14]. Specialists from Recife raised the hypothesis of an association between ZIKV infection in pregnancy and microcephaly. The Brazilian Ministry of Health then established compulsory notification of microcephaly. On 12 November 2015, the Ministry of Health declared a national public health emergency [15]. On 1 February 2016, the World Health Organization (WHO) declared Zika a public health emergency of international concern [16]. At the time, there was no direct scientific evidence of a causal relationship between ZIKV infection during pregnancy and congenital brain defects in fetuses or newborns, although the spatial diffusion of the microcephaly epidemic followed the ZIKV spread paths from an epicentre in the Northeast region towards the south and west in rapid succession (Figure 2).

**Figure 3:**
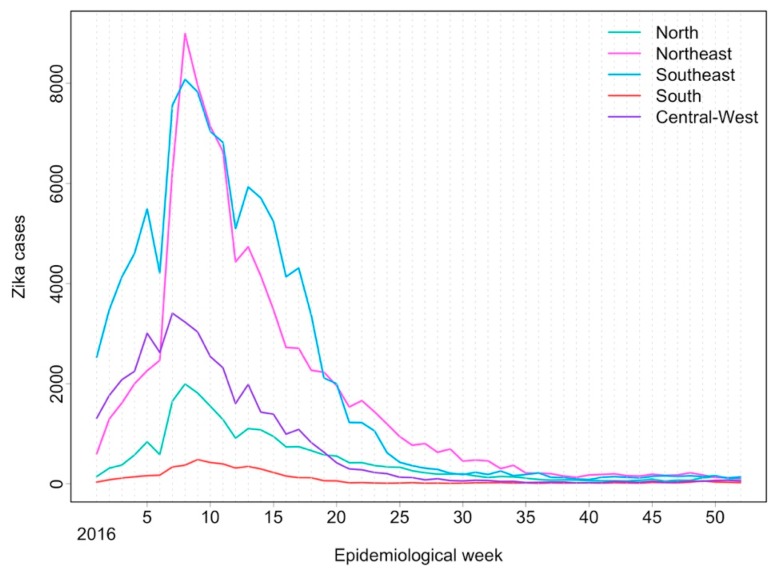
Notified Zika cases for the main regions of Brazil (North, Northeast, Southeast, South, and Central-West) per epidemiological week in 2016. Data obtained from the national notifiable disease information system (SINAN).

**Figure 4:**
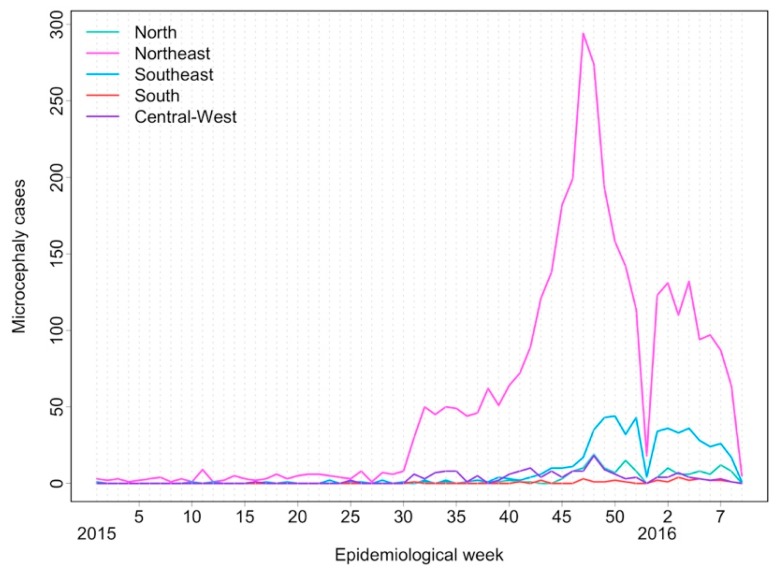
Notified microcephaly cases for the main regions of Brazil (North, Northeast, Southeast, South, and Central-West) per epidemiological week from January 2015 to April 2016. Data obtained from the RESP information system.

Some characteristics of the Brazilian epidemic were helpful in establishing the link between ZIKV infection and microcephaly and other congenital malformations of the central nervous system (CNS) early in the ZIKV epidemic. Firstly, the huge population affected by the infection. In the French Polynesian epidemic, approximately 32,000 presented with the disease among 268,000 inhabitants [17], while in Brazil, more than 200,000 cases were notified by the end of 2016 (see Figure 3) [18]. Secondly, the high reach of the unified public health system, including hospitals in which more than 80% of babies are delivered. The public health obstetricians and neonatologists within this community, dealing with hundreds of childbirths per month, were the first to suspect something was amiss. Thirdly, the close network of practitioners and researchers working within the public health system, including doctors, midwives, epidemiologists, and other academics exchanging information and reporting new findings in a timely fashion. From November 2015, reports of suspected microcephaly cases increased 10-fold within just a few weeks (see Figure 4).

The first case definition of ZIKV infection adopted by the Brazilian Ministry of Health was presented in a protocol for the surveillance and response of ZIKV-related microcephaly [19]. The suspected case definition for pregnant women was broader, including any acute exanthematic disease, unless another cause was identified. The overlap in the space-time epidemiological distribution of Zika, dengue, and chikungunya, and their non-specific clinical manifestations, was challenging for providing accurate diagnosis and reporting. Zika case confirmation included laboratory diagnostics through reverse transcription polymerase chain reaction (RT-PCR). However, this test is not available in most Brazilian states. Further, diagnosis can be problematic due to the limited window of time that viral particles persist in the bloodstream, a large proportion of asymptomatic infections, and the antibody cross-reactivity between ZIKV and other flaviviruses, especially the four dengue virus serotypes, DENV1-4 [13].

The suspected link between ZIKV infection and microcephaly was a highly debated topic in academia and the media. Controversies included the sensitivity and specificity of the cranial measures to define microcephaly [20,21] and alternative causes of the microcephaly epidemic, including the use of Pyriproxifen in drinking-water [22] and a rubella vaccine used in pregnant women. In December 2015, the 49th annual meeting report of the Latin American Collaborative Study of Congenital Malformations concluded that there was not enough evidence to prove the occurrence of a microcephaly epidemic in Brazil, or the causal relationship between ZIKV infection and the congenital syndrome [23]. In fact, the need to be ‘absolutely’ sure about this public health emergency could have impaired the subsequent investigation. The function of public health surveillance systems is to provide actionable information, allowing decision-makers to respond to anomalies in the system as quickly as possible. The scientific controversy resulted in a delayed response, especially for the women and girls suffering the worst impacts of the Zika epidemic [24].

### 2.2. Epidemiological Surveillance and Health Information Systems

Several data sources have proved useful for the epidemiological investigation of ZIKV in Brazil. These include the national notifiable information system (SINAN), as well as information systems on mortality (SIM), newborns (SINASC), and public hospitals discharge (SIH), scaled to the municipality level (5570 municipalities distributed across 27 states).

In November 2015, a new public health events registry (RESP) was created and implemented during the emergency response for notification of cases of microcephaly, other congenital anomalies, and fetal loss, based on the monitoring of pregnant women and their newborn babies [25]. An updated protocol for ZIKV-related microcephaly and other CNS disorders related to congenital infections was published in March 2016 to describe the epidemiological patterns [26]. However, Zika itself did not become a notifiable disease on SINAN until 17 February 2016 [27]. By the end of 2016, approximately 17% of women with ZIKV infection were pregnant (96,494 cases reported in women of a childbearing age) [28]. However, important information, such as education level and race/skin colour, were missing in most of the notification forms, making it difficult to later evaluate risk by socio-economic situation.

Recent studies have explored the use of these health information systems to detect changes in disease patterns that could be attributable to ZIKV. Barcellos et al. [29] detected a four-fold increase in congenital malformations of the nervous system in the SIH in the Northeast region of Brazil from mid 2014 to early 2016. De Oliveira et al. [30] used both the specific RESP and the SINASC, linked together using individual information, such as full name, available only for ethically approved projects. The authors found large variations in ZIKV-related microcephaly incidence between different regions in 2015–2016, varying from almost 5 to 50 per 10,000 live births. These two studies highlight the importance of timely access to secondary data and careful use of record linkage processes [31]. While these analyses present a promising approach, other experiments should be considered for disease surveillance, even when the disease is not yet of public health importance [32]. For example, public-health-integrated surveillance systems, instead of disease-based ones, have been recommended to better forecast, detect, and respond to disease outbreaks and other events of public health significance [33].

## 3. ZIKV Transmission

### 3.1. Vector-Borne Transmission

*Aedes aegypti* is considered to be the main vector of ZIKV in urban and suburban areas together with *Aedes albopictus* [34,35,36]. *Ae. aegypti* and *Ae. albopictus* share similar larval habitats, co-occuring in both natural and artificial containers. Both vectors are invasive species in many regions of the world, including Brazil [37,38], and are closely associated with the human peridomestic environment [39,40,41]. *Ae. aegypti* is a diurnal mosquito, highly anthropophilic and endophilic, that consumes blood multiple times per gonothophic cycle, a behaviour that reinforces its potential as an arbovirus vector. In contrast, *Ae. albopictus* shows an eclectic feeding behaviour, preferentially feeding and resting in the peridomicile, and is more common in vegetated, rural, and urban forest transition habitats, especially where sympatric with *Ae. aegypti* [37,38,40,42]. *Ae. aegypti* inhabits most of Latin America east of the Andes and north of Argentina, reaching the southeast of the United States of America (USA). In Brazil, the most infested country on the continent, the vector is present in all states and in 4834 of the 5570 municipalities. *Ae. albopictus* distribution extends even further, almost reaching the Great Lakes in the USA, due to its tolerance of milder and lower temperatures [43]. ZIKV has been detected in field-caught *Ae. aegypti* mosquito specimens [44,45,46], such as those from the densely urbanised slums of Rio de Janeiro [46].

The ability of different vector species to transmit ZIKV and other arboviruses is sensitive to a combination of factors, including vector population, virus strain, and environmental factors, such as ambient temperature and diurnal temperature range. Both venereal and vertical transmission in mosquitoes is possible, providing a potential mechanism for the virus to survive in adverse environmental conditions [47,48,49]. A recent study evaluated the vector competence of *Ae. aegypti* and *Ae. albopictus* populations from across the Americas for the ZIKV Asian genotype. High infection but lower disseminated infection and transmission rates was observed for both species, suggesting low competence to transmit ZIKV [35]. In contrast, another study showed that orally exposed *Ae. aegypti* mosquitoes were highly competent, with transmission rates of up to 73% for ZIKV, 21% for chikungunya virus (CHIKV), and 12% of mosquitoes transmitting both viruses in one bite [50]. Other studies have also shown simultaneous transmission of alphaviruses and flaviviruses in *Ae. albopictus* and *Ae. aegypti* [51,52,53].

Several laboratory experiments have reported significant differences in ZIKV susceptibility between mosquito species, including *Ae. aegypti*, *Ae. albopictus*, and *Culex quinquefasciatus* [35,54,55,56]. A study conducted in Brazil detected ZIKV in the midgut, salivary glands, and saliva of laboratory-reared *Cx. quinquefasciatus* females [56]. Another study in China showed the ability of *Cx. quinquefasciatus* to become infected and transmit ZIKV under laboratory conditions [57]. In contrast, field populations of *Cx. quinquefasciatus* collected in four different sites in Rio de Janeiro, Brazil, were not able to transmit ZIKV under experimental conditions. After an experimental oral infection, the tested populations failed to present dissemination or transmission three weeks post-exposure [58]. Other experimental studies found no species of the genus *Culex* capable of transmitting ZIKV [59,60,61]. These studies show differences in susceptibility of *Culex* vectors to ZIKV, indicating a complex interaction between the virus and different mosquito species. As discussed elsewhere [62,63], these experiments should be replicated, preferably at the local level, in places that are already endemic or receptive to ZIKV transmission.

ZIKV originated and continues to circulate in a sylvatic transmission cycle between nonhuman primate hosts and arboreal mosquitoes in Africa and Asia [64,65]. In tropical Africa, ZIKV strains have been isolated from different mosquito species, including *Aedes furcifer*, *Aedes taylori*, and *Aedes luteocephalus* [66]. A survey in Senegal detected the presence of Zika virus RNA by RT-PCR in ten species from the genus *Aedes*, as well as from *Mansonia uniformis*, *Anopheles coustani*, and *Culex perfuscus* [64]. A modelling study revealed a high probability of the establishment of sylvatic ZIKV in the Americas with a focus on Brazil, which has multiple species of primates and mosquitoes potentially capable of ZIKV transmission [65]. ZIKV has now been detected in neotropical primates in Brazil [67]. In other parts of the world, ZIKV antibodies have been found in wild and domestic animals, including sheep, cows, rodents, and bats [68]. More research is needed to better understand the potential for nonhuman primates and other animals to be ZIKV reservoirs and the risk they pose for ZIKV transmission to humans.

### 3.2. Non-Vector-Borne Transmission

ZIKV can also be transmitted via sexual contact and blood transfusions. Studies demonstrated the presence and persistence of ZIKV in the male and female genitourinary tract by testing sperm, urine, and vaginal secretions from infected patients over extended periods of time [69,70,71,72]. However, the maximum duration of infectivity of semen and female genital fluids and the impact of ZIKV infection in sexual transmission in areas with or without vector-borne transmission is not known. ZIKV has also been detected in breast milk [73,74], although the transmission of ZIKV through breast milk has not yet been reported [75]. The transmission of ZIKV by blood transfusion was reported in Brazil in 2016 [76]. However, as with sexual transmission, transfusion-transmitted infection is difficult to prove and measure in mosquito-exposed endemic areas [71].

## 4. Clinical Manifestations of ZIKV

Besides the mild febrile exanthematic clinical manifestation of ZIKV infection, itching is an important symptom during the acute period. Two main severe neurological ZIKV-related complications have been identified: Guillan–Barré Syndrome (GBS), a rare condition in which a person’s immune system attacks the peripheral nerves, also described in the 2013 French Polynesia Zika outbreak [77], and microcephaly, the more severe end of a spectrum of birth defects [78], which is sometimes referred to as Congenital Zika Syndrome (CZS) [79].

The detection of ZIKV in the amniotic fluid of pregnant women with fetuses with microcephaly and in fetal brain tissue were the first pieces of evidence that supported the hypothesised link between ZIKV infection during pregnancy and severe fetal/newborn sequelae [80]. Definitive epidemiological evidence was initially derived from two studies in Brazil: a cohort of pregnant women in Rio de Janeiro, which detected congenital abnormalities in 12 fetuses from 42 ZIKV-positive women (29%), but none in 16 ZIKV-negative women [81]; and a case-control study conducted in Recife, in which 32 microcephaly cases as well as 64 control neonates without microcephaly were enrolled. Forty-one percent of microcephaly cases and none of the controls had laboratory evidence for ZIKV infection [82]. Microcephaly and other birth defects in ZIKV-infected mothers had not been recognised previously in other parts of the world. Possible reasons include herd immunity, a lack of diagnostics and surveillance in epidemic areas, and the possibility of contemporary ZIKV strains acquiring adaptive mutations to become more virulent to the human fetal brain [83]. The evidence for causality between ZIKV infection and birth defects has been extensively reviewed [84,85]. The risk of vertical transmission exists throughout pregnancy for both symptomatic and asymptomatic mothers [86], although the exact risk of microcephaly and other birth defects following ZIKV infection is unknown. Medium- and long-term follow-up with careful standardised evaluations are needed to determine overall outcomes in the exposed infants, including rates of developmental delay, hearing and visual impairments, seizure and feeding disorders, as well as later outcomes, such as learning disabilities.

## 5. Control and Prevention Strategies

### 5.1. Vector Control

Currently, there are no viable ZIKV-specific vaccines or therapies available. Therefore, vector control is the primary method for the control and prevention of mosquito-borne diseases, such as ZIKV [87,88]. Several control strategies have been implemented in urban areas of Brazil, but there is no evidence that any recent vector-control interventions, such as mechanical and biological control or the use of larvicide and insecticides, have had any significant effect on DENV transmission [89,90]. The House Index—a household larval survey—is typically used to guide vector control efforts, but this approach has been found to be ineffective and expensive [89]. Both *Aedes* vectors are widely distributed and numerous in most small and large cities [38,46]. Rapid human population growth, uncontrolled urbanization, including slum settlements with inadequate infrastructure and piped water, and insecticide resistance, have made it very difficult to reduce *Ae. aegypti* populations to safe levels [90,91].

In Brazil, several new approaches are being implemented to change paradigms in *Ae. aegypti* control. In Recife, a massive trap intervention was implemented for two years with population suppression of about 90% [92]. Sterile [93], transgenic mosquitoes [94] and mosquitoes bearing *Wolbachia* [95,96] are being released under field-controlled conditions to better evaluate their impact on local vector populations and arbovirus transmission. There are promising results regarding transgenic mosquitoes, with sustained release reducing field populations of *Ae. aegypti* by 81–95% [97]. Mosquito-disseminated Pyriproxyfen is another novel control strategy being used in Brazil, with promising results regarding the reduction of field populations of *Ae. aegypti* and the predicted number of dengue cases [98,99].

### 5.2. Mathematical Modelling to Guide Interventions

Mathematical and computational modelling approaches, integrating demographic, human mobility, climate, socio-economic, and mosquito density data, have been developed to understand the spread and magnitude of ZIKV epidemics in the Americas [100], estimate the international spread of the virus from Brazil [101], and project the near-term risk of ZIKV infection and the associated congenital syndrome [102]. However, the interpretation of model results is limited by many factors, including the unknown rate of asymptomatic infection, herd immunity, and inconsistencies in case reporting [103]. Some ZIKV predictions have been extrapolated from empirical models of DENV [104]. A recent study used a two-vector basic reproduction number model to quantify the impact of climate variability on ZIKV and other arbovirus transmission [105]. The authors found that temperature conditions at the beginning of 2015, related to a strong El Niño event, were exceptionally conducive for mosquito-transmitted diseases and could have been successfully predicted at least 1 month in advance for several high-risk ZIKV zones, including the epicentre of the epidemic in Northeast Brazil. Previous studies have used real-time seasonal climate forecasts to produce dengue early warnings for Brazil [106,107]. A combination of forecast climate and seroprevalence survey data could improve predictions of the timing and magnitude of outbreaks of multiple arboviruses, including ZIKV [108]. An important question for the modelling community is why the epidemic in the Americas seems to have run its course. Herd immunity is thought to have played a major role [12]. However, the extent to which other factors, such as vector control, modified human behavior, and reporting practices, may have contributed to the apparent decline in ZIKV cases is not well-understood [109]. Well-formulated predictive models are needed to help policy-makers anticipate when and where the next epidemic of ZIKV or another emerging infectious disease is likely to hit, to plan vaccination campaigns, and target innovative vector control technologies to the most at risk areas [110].

### 5.3. Birth Control

Although data is limited, guidance is needed for couples who are exposed to ZIKV and planning pregnancy. Advice from the Centers for Disease Control and Prevention (CDC) is based on a study that detected ZIKV RNA in semen for as long as 188 days after symptom onset [71]. The guidance states that women and men who reside in areas of transmission and experience symptoms of Zika disease should be tested for ZIKV infection. Men with results that indicate recent ZIKV or another unspecified flavivirus infection should wait at least six months from symptom onset before attempting conception with their partner, while women should wait at least eight weeks from symptom onset before attempting to conceive [111].

In 2016, a protocol was published on reproduction rights and pre-natal, delivery, and puerperal care in response to microcephaly and other CNS disorders [112], but the report did not mention abortion, which is illegal in Brazil. The number of live births dropped by 15% between September and December 2016 in Rio de Janeiro compared to the previous year [113]. Possible reasons include delayed pregnancy, ZIKV increasing the risk of miscarriage and fetal death, voluntary abortion, or pregnancy interruption [114]. However, many women in Brazil have unwanted pregnancies and are at risk of unsafe abortion, which needs to be urgently addressed by the public health system [115,116,117,118].

## 6. Social and Economic Impact

Congenital Zika Syndrome (CZS), including severe microcephaly and a range of other birth defects, has wide-ranging impacts for the child, the family, and society as a whole. Children with CZS are likely to have a broad range of intellectual, physical, and sensory impairments, which will be life-long. CZS is new, but childhood disability is not, and its impacts are well-established. Extensive evidence shows that children with disabilities face a range of exclusions and difficulties [119]. For instance, childhood disability is strongly linked to poverty [120], malnutrition [121], vulnerability to violence [122], poor health [123], and exclusion from schools [123]. These difficulties are likely to continue into the future, as adults with disabilities are less likely to be employed, and more likely to face poverty and wide-ranging social exclusion [119,120].

Children with CZS are disproportionately born to women and girls of low socio-economic status, who are usually the main caregivers [115]. The negative impacts of being a carer are now well-recognised [124], including vulnerability to anxiety and depression [125] and deepening poverty [126]. The impact of CZS extends beyond the mother alone, affecting the whole family. The arrival of a child with disabilities into a family can often be a trigger for fathers to abandon the family. It may also negatively impact siblings, as less care and attention can be dedicated to them [115].

Looking beyond the family, there are wider societal impacts of CZS. The epidemic may have affected child-bearing decisions of young adults, as well as cause concern among pregnant women. Health professionals may face the strain of having to deal with a new condition, the trajectory of which is unknown, and for which services are not widely available. The impact may be experienced on a broader national level, as the health system comes under pressure and as the economic consequences for affected families coalesce at the societal level. Overall, these factors may create further social and economic concerns within Brazil, which is a country currently experiencing widespread political and economic problems.

Solutions should be targeted to address the full range of impacts of CZS. Currently, most efforts are being made to meet the medical needs of the affected children. A large focus is on the children with microcephaly, while those less severely affected may experience gaps in their treatments, as their diagnosis is delayed or they are not prioritised for services. So far, no follow-up of otherwise healthy children with postnatal ZIKV infection or exposure has been made. Therefore, developmental complications should be tracked in all potentially exposed children of the “Zika generation” [111,127,128].

Financial support grants have been made available by the government and should be given to most poor families of children with disabilities, yet those with microcephaly appear to be prioritised while others with less severe disabilities may be missing out [115], despite the high cost of treatment [126]. In different parts of Brazil, some support programmes have been implemented, often established by parents of children with CZS or else by childhood disability programmes. However, these programmes rarely address the comprehensive needs of families and coverage is patchy. Therefore, there is likely to be a large unmet need for psychosocial support. Healthcare professionals and family members may also need further training in how to address the medical and broader need of these children. In the longer-term, the children will need support to be included in education, employment, and society in general, in fulfilment of their fundamental rights [129].

## 7. Knowledge Gaps

**Figure 5:**
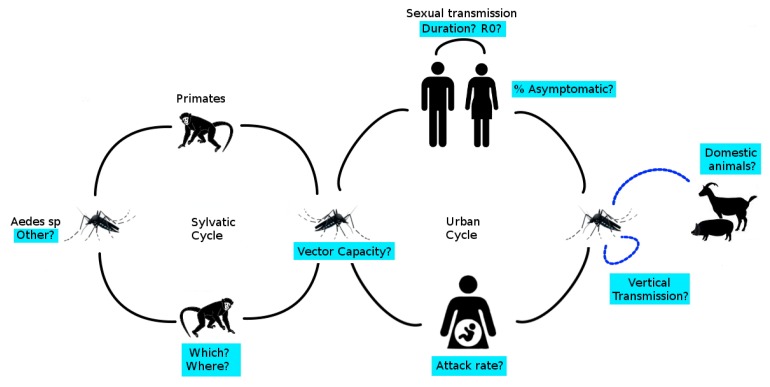
Summary of known epidemiological features of ZIKV. Unresolved questions are highlighted in blue. R_0_ is the basic reproduction number (the expected number of secondary cases produced by a single infection in a completely susceptible population).

This article highlights some of the important scientific advances that have been made since the emergence of ZIKV in Brazil. However, some gaps in our knowledge of the epidemiology, clinical evolution, virology, and biology of ZIKV infections remain, including potential domestic and wild animal reservoirs, amplification hosts, vector capacity of various species, and alternative non-vector transmission routes (see Figure 5). Unresolved areas of research include: the factors which led to its explosive emergence; the impact of previous flavivirus infection on the severity of ZIKV infections; the duration of ZIKV infection immunity; and the level of background immunity required to prevent emergence or re-circulation of ZIKV in the future.

Substantial evidence now exists on the association between materno-fetal transmission of ZIKV and congenital CNS malformations. However, several important issues remain unresolved, including: the transmission rate from infected pregnant women to fetuses; the frequency of infected fetuses that will develop malformations; and the long-term outcome of infected neonates without detectable abnormalities at birth. It is still not known if apparently unaffected children whose mothers had ZIKV in pregnancy will develop normally or develop complications that will only become evident in years to come.

## 8. Conclusions

The ZIKV epidemic in Brazil has ended for the time being, and the disease and associated complications are no longer considered a public health emergency of international concern [130]. However, the outbreak will have long-lasting social and economic impacts, especially for the families impacted by CZS. The Brazilian Ministry of Health has pledged to maintain actions to confront the *Aedes* mosquito and guarantee access to health services for the affected populations [131]. However, the national response to the Zika crisis has largely focused on household-level mosquito elimination efforts, such as cleaning water storage containers, eliminating standing water, and spraying. These efforts are often futile without addressing systemic problems with public infrastructure, such as limited access to piped water and poor sanitation [126]. Such conditions provide ideal mosquito habitats, which exacerbated the recent ZIKV outbreak, as well as outbreaks of other arboviruses in Brazil [115].

Computational models can help understand the role of multiple ZIKV transmission risk factors at different spatial and temporal scales, such as climate, human mobility, socio-economic status, asymptomatic infections, and background immunity. They have the potential to assist decision-makers in understanding where ZIKV will spread next and when the next ZIKV epidemic might occur. However, effective ZIKV modelling initiatives depend upon flexible and open-access surveillance systems that are capable of detecting new health threats and improved laboratory diagnostic tools. A continued and intensified international and interdisciplinary response is needed to improve our ability to anticipate, control, and mitigate the risk of ZIKV, other reemerging arboviruses, and new public health threats yet to emerge.
